# Traumatic events during childhood and its risks to substance use in adulthood: an observational and genome-wide by environment interaction study in UK Biobank

**DOI:** 10.1038/s41398-021-01557-7

**Published:** 2021-08-20

**Authors:** Shiqiang Cheng, Yan Wen, Li Liu, Bolun Cheng, Chujun Liang, Jing Ye, Xiaomeng Chu, Yao Yao, Yumeng Jia, Om Prakash Kafle, Feng Zhang

**Affiliations:** Key Laboratory of Trace Elements and Endemic Diseases of National Health and Family Planning Commission, School of Public Health, Health Science Center, Xi’an Jiaotong University, Xi’an, China

**Keywords:** Addiction, Clinical genetics

## Abstract

We aimed to explore the underlying genetic mechanisms of traumatic events during childhood affecting the risks of adult substance use in present study. Using UK Biobank cohort, linear regression model was first applied to assess the relationships between cigarette smoking and alcohol drinking in adults with traumatic events during childhood, including felt hated by family member (41,648–111,465), felt loved (46,394–124,481) and sexually molested (47,598–127,766). Using traumatic events as exposure variables, genome-wide by environment interaction study was then performed by PLINK 2.0 to identify cigarette smoking and alcohol drinking associated genes interacting with traumatic events during childhood. We found that the frequency of cigarette smoking was significantly associated with felt hated by family member (coefficient = 0.42, *P* < 1.0 × 10^–9^), felt loved (coefficient = −0.31, *P* < 1.0 × 10^–9^) and sexually molested (coefficient = 0.46, *P* < 1.0 × 10^–9^). We also observed weaker associations of alcohol drinking with felt hated by family member (coefficient = 0.08, *P* = 3.10 × 10^–6^) and felt loved (coefficient = −0.06, *P* = 3.15 × 10^–7^). GWEIS identified multiple candidate loci interacting with traumatic events, such as CTNNA3 (rs189142060, *P* = 4.23 × 10^–8^) between felt hated by family member and the frequency of cigarette smoking, GABRG3 (rs117020886, *P* = 2.77 × 10^–8^) between felt hated by family member and the frequency of alcohol drinking. Our results suggested the significant impact of traumatic events during childhood on the risk of cigarette smoking and alcohol drinking.

## Introduction

Smoking and drinking behaviors are serious public health concerns, which can lead to two common substance dependence, nicotine dependence and alcohol dependence. Excessive cigarette and alcohol consumption are leading causes of preventable death [1, 2]. There are ~976 million smokers in the world [3]. In 2017, 20% of adults were heavy drinkers, and it is expected that the proportion will increase to 23% globally by 2030 [4]. In 2015, about 6.4 million people died from smoking globally, among which, chronic respiratory diseases (20.5%), cancers (27.6%) and cardiovascular diseases (41.2%) are the three main causes of age standardized disability-adjusted life-years (DALYs) attributing from smoking [5]. According to a systematic analysis, alcohol use disorders is the most common of all substance use disorders, with an estimated 100 million cases worldwide in 2016 [6]. The global DALYs resulted from alcohol use were highest in cancers, injuries, and cardiovascular diseases [6].

Smoking and drinking behaviors are attributable to multiple factors with significant genetic effects [7]. Recently, genome-wide association studies (GWAS) have identified multiple loci involving with nicotine and alcohol dependence [1, 8]. For example, some of the loci involves of genes CHRNA3, CHRNB4, and CHRNA5 that encode the receptor subunits of neuronal nicotinic acetylcholine [8]. More recently, the researchers have established more than 500 genetic variants contributing to different stages of alcohol use and nicotine use, including the initiation, heaviness and cessation of this two behaviors [9]. However, many of those studies focused on the impact of individual genetic variants, rather than their interplay with environmental risk factors.

It has been demonstrated that environmental, genetic, and psychological factors and their interactions all contribute to the smoking and drinking behaviors [10, 11]. However, it has been difficult to define the nature of the interactions between these factors. Genome-wide by environment interaction study (GWEIS) is a prefunding tool to explore disease associated genetic variations that interact with environmental risk factors. For example, in a GEWIS of stressful life events in African Americans, a significant signal was identified for depressive symptoms [12]. Gong et al. have suggested that genome-wide significant interaction between alcohol consumption and genetic factors contributed to reveal the etiology of colorectal cancer and distinguish subgroups [13].

Traumatic events during childhood have a great impact on the development of the substance use. For example, Jun et al. determined associations between both accumulation and severity of early initiation smoking and the risk of abuse among girls and the extent of familial emotional support has a protective effect on smoking [14]. Childhood sexual assault has been demonstrated to be related to smoking and other kind of substance abuse [15, 16]. With regard to alcohol misuse, childhood sexual abuse has consistently been shown a risk factor for heavy drinking and alcohol-related problems [17, 18].

We aimed to explore the effects of traumatic events during childhood on adult substance use in UK Biobank cohort and to investigate its underlying genetic mechanisms. First, we estimated the association of traumatic events, such as felt loved as a child, felt hated by family member as a child and sexually molested as a child with the amount of smoking and drinking through a linear regression model. GWEIS was then applied to explore the genetic variation interaction between those traumatic events and the frequency of cigarette smoking and alcohol drinking.

## Materials and methods

### UK Biobank dataset

The UK Biobank study is a prospective cohort included health, hospital-records and genetic data from more than 0.5 million participants [19]. UK Biobank has electronic signed consent from the study participants and ethical approval was obtained from Northwest Multi-center Research Ethics Committee. We used the imputed genotype dataset released by UK Biobank in July 2017. Subjects were excluded if the self-reported gender were inconsistent with the genetic gender, or were genotyped but not imputed or withdraw their consents.

DNA samples of all participants in the UK Biobank were genotyped using either the Affymetrix UK Biobank Axiom (825,927 markers) array or Affymetrix UK BiLEVE (807,411 markers) [20]. SNPs were imputed by IMPUTE2 against the reference panel of the, 1000 Genomes, UK10K projects, and Haplotype Reference Consortium. The details regarding these data are available elsewhere [21]. The current research has been performed under the Application Number 46478. The authors thank all UK Biobank participants and researchers who contributed or collected data.

### Phenotypes definition

Traumatic events during childhood, including felt loved as a child, felt hated by family member as a child and sexually molested as a child, were collected from the response to the experiences during childhood from the UK Biobank on-line “Thoughts and Feelings” mental health questionnaire: “When I was growing up …” a) I felt loved, c) I felt that someone in my family hated me and d) Someone molested me (sexually) by choosing “Never true (0)”, “Rarely true (1)”, “Sometimes true (2)”, “Often (3)”, “Very often true (4)”, and “Prefer not to answer (−818)”. The individuals whose answers are “Often (3)” and “Very often true (4)” were treated as cases, and whose answers are “Never true (0)” and “Rarely true (1)” were treated as controls respectively. The subjects whose answers are “Sometimes true (2)” and “Prefer not to answer (-818)” were excluded in this study.

The frequency of cigarette smoking and alcohol drinking of each were collected by the amount of smoking and drinking of self-report respectively. The cigarette smoking was coded as 0 if ever-smoking status was also 0, otherwise, the maximum number of reported past or current cigarettes (or pipes/cigars) consumed per day was used. Similarly, the alcohol drinking was coded as 0 if ever-drinking status was 0, otherwise, the average amount of different types of alcohol per week the weekly beverage phenotype for these individuals were used. Those who reported drinking less than once a week were asked the average amount of different types of alcohol per month. For these individuals, the total amount of alcohol consumed each month was added up and divided by 4 to get about the amount consumed per week. All variables were standardized to have mean 0 and variance 1 before further analysis. Detailed information of study subjects was provided in Table 1.

**Table 1:** Basic characteristics of study subjects from UK Biobank cohort.

		Felt loved as a child	Felt hated by family member as a child	Sexually molested as a child
Alcohol freq per week	*N*	41,648	46,394	47,598
	Sex (female)	22,632	24,933	25,545
	Age^a^	56.17 (7.70)	56.21 (7.68)	56.15 (7.69)
Smoke freq per day	*N*	111,465	124,481	127,766
	Sex (female)	63,808	70,489	72,218
	Age^a^	55.89 (7.73)	55.92 (7.72)	55.84 (7.73)

^a^Age was described as mean (standard deviation).

### Statistical analysis between traumatic events and substance use

The associations between traumatic events during childhood and substance use were estimated using a multivariable linear regression model. The exposures variables were traumatic events during childhood including felt loved as a child, sexually molested as a child and felt hated by family member as a child, and the outcome variables were substance use including the frequency of cigarette smoking and alcohol drinking. Sex, age and the first ten principle components (PCs) of population structure were adjusted as covariates. Beta coefficient with 95% confidence intervals (CI) and *p* values were calculated by the multivariable linear regression model. All statistical analyses were conducted by R 3.5.1 (https://www.r-project.org/).

### Genome-wide by environmental interaction analysis

GWEIS was conducted to explore the interaction effects between SNP and traumatic events on the risk of substance use in UK Biobank cohort. The outcomes variables, including the frequency of cigarette smoking and alcohol drinking were adjusted by age, sex, and the first ten PCs of population structure. The allelic dosage additive effect model of PLINK 2.0 was selected in this study [22]. The SNPs with call rate <0.95, Hardy Weinberg equilibrium testing *P* value < 0.001 and minor allele frequencies <0.01 were excluded for variations quality-control [22]. A significance threshold was set at *P* = 5.0 × 10^−8^ for genome-wide by environment interaction effects. The Manhattan plots were generated using the “CMplot” R script (https://github.com/YinLiLin/R-CMplot).

## Results

### Association between traumatic events and substance use

**Fig. 1:**
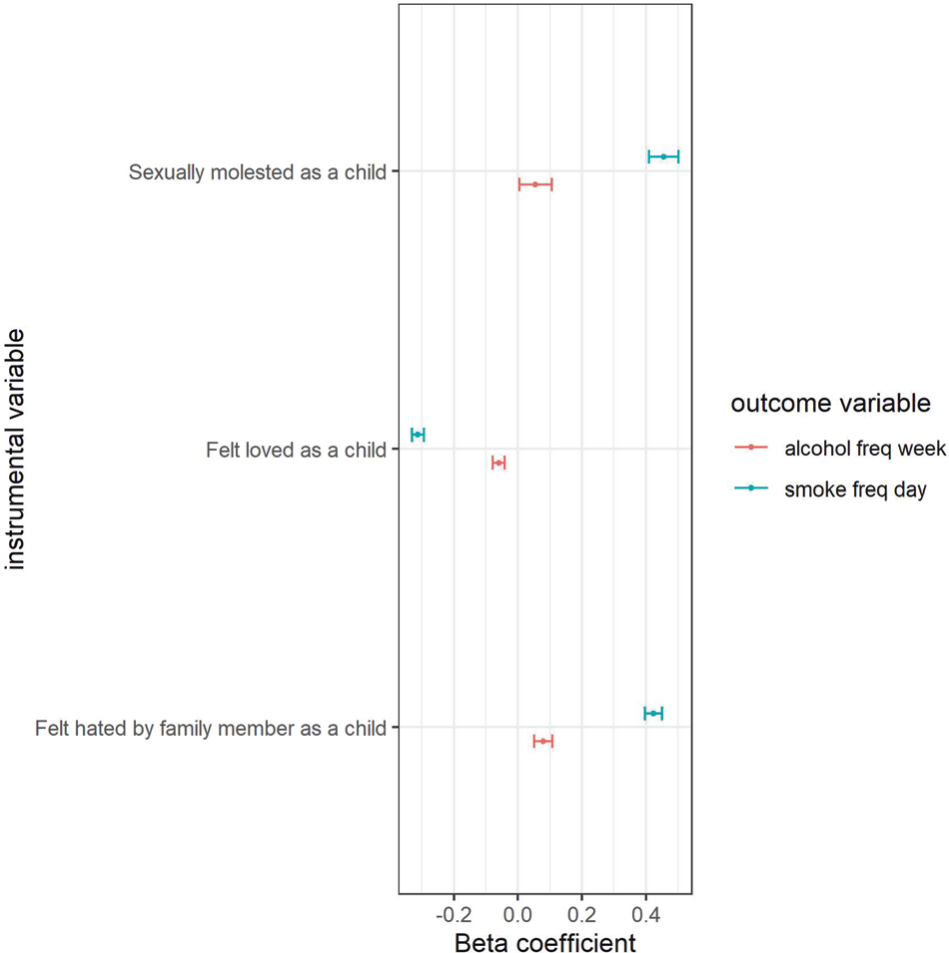
Association between traumatic events during childhood and substance use in UK Biobank population. *The *x*-axis refers to beta coefficient (B). The *y*-axis represents the instrumental variables. Points display the B and 95% CIs (error bars) of B. Detail information was showed in Supplementary Table 1.

We found that the frequency of cigarette smoking was significantly associated with felt hated by family member as a child (coefficient = 0.42, *P* < 1.0 × 10^–9^), felt loved as a child (coefficient = −0.31, *P* < 1.0 × 10^–9^) and sexually molested as a child (coefficient = 0.46, *P* < 1.0 × 10^–9^). The frequency of alcohol drinking was significantly associated with felt hated by family member as a child (coefficient = 0.08, *P* = 3.10 × 10^–6^) and felt loved as a child (coefficient = −0.06, *P* = 3.15 × 10^–7^). The detailed association were provided in Fig. 1 and Supplementary Table 1.

### Interactions between individual SNPs and traumatic events

**Fig. 2:**
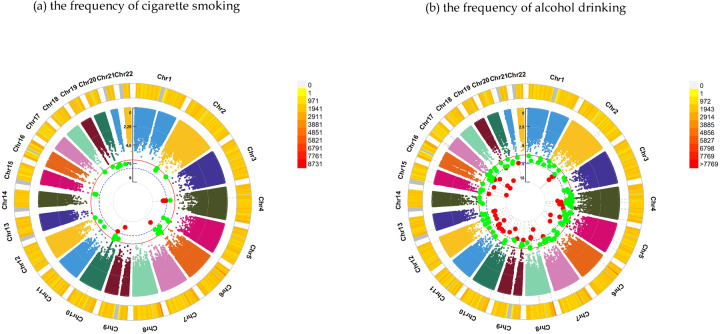
Chromosomal regions interacting with felt hated by family member as a child for substance use. *From the center, the circos depicts the −log_10_ *P* values of each variant. Red plots represent the *P* value < 5 × 10^−8^ and green plots represent *P* value < 5 × 10^−7^. The plots were generated using the “CMplot” R script (https://github.com/YinLiLin/R-CMplot).

For felt hated by family member as a child, GWEIS identified 69 candidate loci, such as CTNNA3 (rs189142060, *P* = 4.23 × 10^–8^) for the frequency of cigarette smoking and DLGAP2 (rs138412709, *P* = 1.64 × 10^–9^) and GABRG3 (rs117020886, *P* = 2.77 × 10^–8^) for the frequency of alcohol drinking. More details were provided in Tables 2, 3, Fig. 2 and Supplementary Tables 2, 3.

**Table 2:** The summary of genetic variants interacting with traumatic events during childhood in the frequency of cigarette smoking.

Traumatic events during childhood	Variation ID	Target gene	*P* value
Felt hated by family member	rs189142060	CTNNA3	4.23 × 10^–8^
	rs72779234	MYMK	6.81 × 10^–9^
Felt loved	rs73009056	GRM1	1.53 × 10^–8^
	rs80005225	LSP1	4.31 × 10^–9^
	rs143458035	MIPOL1	8.28 × 10^–9^
Sexually molested	rs200062414	CCSER1	5.35 × 10^–9^
	rs116618591	COL18A1	3.23 × 10^–9^
	rs17115257	DAB1	5.35 × 10^–9^
	rs75052594	ECT2	3.23 × 10^–9^
	rs114730935	EHHADH	5.35 × 10^–9^
	rs77983918	MATN2	3.23 × 10^–9^
	rs183288119	NRXN3	1.34 × 10^–8^
	rs76808343	PAPPA2	3.72 × 10^–8^
	rs62186523	RAD21L1	1.60 × 10^–8^
	rs552868666	RIC1	2.04 × 10^–8^
	rs151099034	RIMS2	3.15 × 10^–8^
	rs17825787	SERPINA12	1.83 × 10^–8^
	rs116618591	SLC19A1	3.23 × 10^–9^
	rs115349299	SORBS2	1.65 × 10^–8^

**Table 3:** The summary of the genetic variants interacting with traumatic events during childhood in the frequency of alcohol drinking.

Traumatic events during childhood	Variation ID	Target Gene	*P* value
Felt hated by family member	rs118114209	C8orf34	1.57 × 10^–8^
	rs186204465	CEP112	1.33 × 10^–10^
	rs61738833	CS	7.66 × 10^–9^
	rs542449847	DCK	4.46 × 10^–9^
	rs138412709	DLGAP2	1.64 × 10^–9^
	rs75210337	GABBR2	3.01 × 10^–8^
	rs117020886	GABRG3	2.77 × 10^–8^
	rs28549240	HERC3	1.36 × 10^–8^
	rs61937726	KSR2	7.24 × 10^–9^
	rs183497996	LRRC4C	1.84 × 10^–8^
	rs542449847	MOB1B	4.46 × 10^–9^
	rs72811298	MYOCD	4.38 × 10^–8^
	rs72844075	OSBPL5	2.14 × 10^–8^
	rs147084289	PCDHG@ Gene	1.36 × 10^–9^
	rs116708930	PLA2R1	4.66 × 10^–9^
	rs492553	POLR1D	7.50 × 10^–9^
	rs77261378	PTPRD	3.44 × 10^–8^
	rs534525861	RDH10-AS1	2.17 × 10^–8^
	rs145009935	SFMBT2	1.91 × 10^–9^
	rs117358906	TANC2	9.77 × 10^–9^
Felt loved	rs79187523	AKAP6	2.01 × 10^–8^
	rs116573968	ANXA5	4.61 × 10^–8^
	rs4752622	ATE1	4.31 × 10^–9^
	rs11592430	CCDC3	3.73 × 10^–8^
	rs111458598	CDK12	2.91 × 10^–8^
	rs114438632	CPNE4	4.57 × 10^–8^
	rs181625218	DACT1	1.67 × 10^–8^
	rs34892827	DST	5.34 × 10^–10^
	rs4532987	FAM21EP	4.94 × 10^–8^
	rs150929669	GUCY1A1	3.27 × 10^–8^
	rs78511804	KIAA0586	8.36 × 10^–9^
	rs75774241	LHFPL6	2.36 × 10^–8^
	rs6530964	LONRF1	1.02 × 10^–8^
	rs1784414	MMP20	3.49 × 10^–8^
	rs79795728	NTN1	3.08 × 10^–9^
	rs78087962	NUP93	1.74 × 10^–9^
	rs17010387	PARG	3.71 × 10^–8^
	rs62522696	PREX2	2.87 × 10^–9^
	rs150200887	RBMS3	2.06 × 10^–8^
	rs10412986	RELB	1.42 × 10^–8^
	rs9787488	RPL5P25	4.95 × 10^–8^
	rs147222280	SLC35F4	2.09 × 10^–9^
	rs79873275	STAC2	1.87 × 10^–8^
	rs78477794	STXBP5	1.10 × 10^–8^
	rs111937104	TOX	4.82 × 10^–8^
	rs142253038	USH2A	3.02 × 10^–9^

**Fig. 3:**
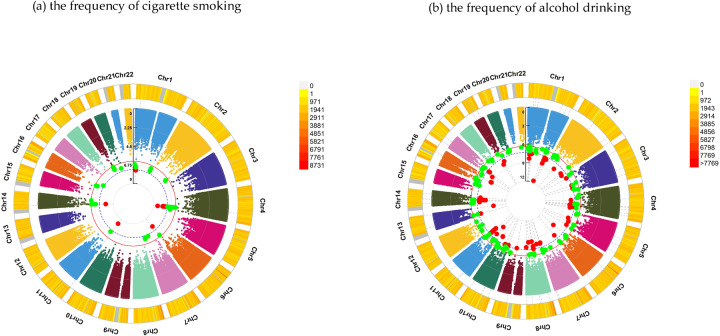
Chromosomal regions interacting with felt loved as a child for substance use. *From the center, the circos depicts the −log_10_ *P* values of each variant. Red plots represent the *P* value < 5 × 10^−8^ and green plots represent *P* value < 5 × 10^−7^. The plots were generated using the “CMplot” R script (https://github.com/YinLiLin/R-CMplot).

For felt loved as a child, GWEIS discovered 164 candidate loci, such as GRM1 (rs73009056, *P* = 1.53 × 10^–8^) for the frequency of cigarette smoking and ATE1 (rs4752622, *P* = 4.31 × 10^–9^) for the frequency of alcohol drinking. More details were provided in Tables 2, 3, Fig. 3 and Supplementary Tables 4, 5.

**Fig. 4:**
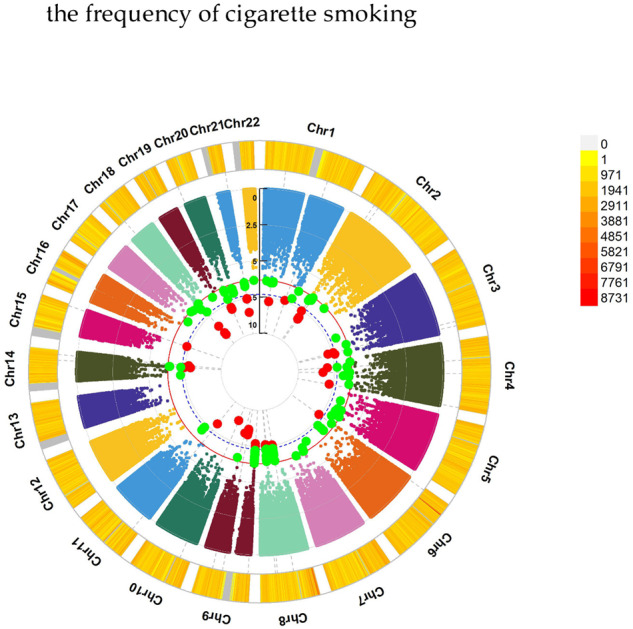
Chromosomal regions interacting with sexually molested as a child for the frequency of cigarette smoking. *From the center, the circos depicts the −log_10_ *P* values of each variant. Red plots represent the *P* value < 5 × 10^−8^ and green plots represent *P* value < 5 × 10^−7^. The plots were generated using the “CMplot” R script (https://github.com/YinLiLin/R-CMplot).

For sexually molested as a child, GWEIS scanned 54 candidate loci, such as NRXN3 (rs564134655, *P* = 1.34 × 10^–8^) for the frequency of cigarette smoking. More details were provided in Table 2, Fig. 4 and Supplementary Table 6.

## Discussion

In the observational study, we found associations between substance use and traumatic events during childhood. It has been reported by previous studies that traumatic events during childhood increases the risk of later substance dependence [15–18]. For example, it has been revealed that exposure to childhood physical and sexual abuse was significantly associated with nicotine dependence and cigarettes smoked per day [23]. The increase in number of childhood adverse events was associated with higher risk of tobacco use and nicotine dependence in alcohol dependence individuals [24]. Jun et al. determined associations between both accumulation and severity of early initiation smoking and the risk of abuse among girls and the extent of familial emotional support has a protective effect on smoking [14]. According to our findings, traumatic events during childhood, such as felt hated by family member as a child and sexually molested as a child, are risk factors for the frequency of drinking and smoking. Familial emotional support, like felt loved as a child is protective against the behavior of smoking and drinking, which are consistent with previous studies.

Less is known about the biological mechanisms of traumatic experiences increasing the risk of substance use. The key point of this study is that we conducted a GWEIS of substance use and identified multiple loci and candidate genes for the regulation of genetic response to traumatic events during childhood, and provided novel clues to help disentangle its underlying etiology. GWEIS identified multiple candidate loci interacting with felt hated by family member as a child for substance use, such as CTNNA3, DLGAP2, and GABRG3. CTNNA3, an alpha-catenin gene, displays clusters of SNPs whose allelic associations with addiction vulnerability [25]. Multiple GWAS have revealed that CTNNA3 is a gene with clusters of SNPs associated with nicotine dependence [26–28]. Uhl et al. demonstrated that variants of CTNNA3 gene may lead to individual differences in the success of smoking cessation [29]. The researchers suggested that CTNNA3 may play its role by encodes a protein involved in extracellular matrix activities and/or cell adhesion, which is essential for the formation and maintenance of synapse [27]. Recently, based on studies of copy number variants, SNPs and a rare compound heterozygous exon deletion, Bacchelli et al. have summarized that CTNNA3 is a candidate gene for the development of ASD [30]. In addition, the alpha-catenin protein is expressed in the pattern of developmentally-regulated cerebral cortex and hippocampus, which indicates that CTNNA3 may mediated cell adhesion in the developing brain [30]. Moreover, CTNNA3 plays an important role in the ependymal cell junctions of the brain ventricles, and its loss could led to compensatory upregulation of CTNNA1 expression [31]. The upstream regions of DLGAP2 gene was identified as a differentially methylated regions (DMR) related to alcohol dependence in an epigenome-wide association study using postmortem tissues [32]. In vitro experiment, methylation at the DMR-DLGAP2 regulated the expression of DLGAP2, and DLGAP2-deficient mice exhibited reduced alcohol consumption in contrast with wild-type controls [32]. The results suggested that DLGAP2 may play its role for genetic and epigenetic factors by controlling alcohol use and dependence [32]. Gamma-aminobutyric acid (GABA) is a major inhibitory neurotransmitter in the human central nervous system. Previous in vitro cell models, animal and human experiment have proved that gamma-aminobutyric acid (GABA) could mediate many of the neurochemical pathways that affect alcohol use and dependence [33]. Dick et al. found that GABA receptor subunits GABRG3 may associated with the risk of alcohol dependence [33]. Moreover, it has been demonstrated that GABRG3 contained a binding sites of Benzodiazepine, which can be used to treat alcoholism and alcohol withdrawal symptoms [34, 35].

For felt loved as a child, we found some candidate loci interacting with felt loved as a child also associated with substance use or brain development. Glutamate metabotropic receptor 1 (GRM1), also known as MGLUR1, is related to the frequency of smoking in this study. Glutamatergic neurotransmission participated in many process of normal brain and can be affected in many neuropathologic conditions [36]. In an animal experiment, the researchers demonstrated MGLUR agonizts can regulate nicotine withdrawal and suggested that LY354740, a MGLUR agonizts, may help alleviate the symptoms associated with nicotine withdrawal during smoking cessation [37]. ATE1 is associated with the frequency of drinking in this study. In an experiment that studies the function of ATE1 in neuronal growth and brain development, Wang et al. indicated that ATE1 is essential for normal development of mouse brain and lack of ATE1 prohibits neurite outgrowth and mislocalizes doublecortin at the growth cones [38].

For sexually molested as a child, NRXN3 were identified to be associated with the frequency of smoking in this study. Neurexins are presynaptic cell adhesion proteins which functioning in the development of GABAergic and glutamatergic synapses [39, 40]. According to a previous study, those synapses involved in key circuits influencing addictive behaviors [41]. NRXN3, a member of the neurexins gene family, has been shown to be related to nicotine dependence [26]. Genotyping association tests have identified three SNPs of NRXN3 gene relate to a lower risk of being a smoker [42]. Novak et al. have suggested that variants in the NRXN3 gene could influence the degree of nicotine dependence in patients with schizophrenia [43].

To the best of our knowledge, this is the first GWEIS of substance use using traumatic events during childhood as exposure variables in a cohort of relatively homogeneous European ancestry. In contrast with GWAS, GWEIS analysis discovered some novel genes that might influence the frequency of cigarette smoking and alcohol drinking. But there is also some limitation of this study. For example, although in the association analysis between traumatic events during childhood and substance use, we have incorporated the factors of age, sex, and ten PCs as covariates. We cannot exclude the effect of other potential confounding factors related to cigarette smoking which do not have data availability in UKbiobank, such as family or marital status, occupation classification, attitude to smoking.

In summary, through observational and GWEIS analyses, this study indicated that traumatic events during childhood may affect the behavior of smoking and drinking and found some candidate genes of their interaction. Identifying the significant gene–environment interactions underlying the behavior of smoking and drinking could help to reduce the incidence and mortality of other complex disease caused by smoking and drinking.

## Supplementary information

Association between traumatic events during childhood and substance dependence.

Interactions between individual SNPs and felt hated by family member as a child in the frequency of cigarette smoking with P <5×10–8.

Interactions between individual SNPs and felt hated by family member as a child in the frequency of alcohol drinking with P <5×10–8.

Interactions between individual SNPs and felt loved as a child in the frequency of cigarette smoking with P <5×10–8.

Interactions between individual SNPs and felt loved as a child in the frequency of alcohol drinking with P <5×10–8.

Interactions between individual SNPs and sexually molested as a child in the frequency of cigarette smoking with P <5×10–8.

## Data Availability

The UKB data are available through the UK Biobank Access Management System (https://www.ukbiobank.ac.uk/). We will return the derived data fields following UKB policy; in due course, they will be available through the UK Biobank Access Management System.
