# Supplementary material for: Traumatic events during childhood and its risks to substance use in adulthood: an observational and genome-wide by environment interaction study in UK Biobank
Source: Transl Psychiatry. 2021 Aug 20;11:431. doi: 10.1038/s41398-021-01557-7 (PMC8379203; doi:10.1038/s41398-021-01557-7)
Supplement: Supplementary file 1 — Association between traumatic events during childhood and substance dependence. [file 41398_2021_1557_MOESM1_ESM.docx]

**Table S1. Association between traumatic events during childhood and substance dependence.**

| **Instrumental variable** | **Outcome variable** | **Beta (95%CI)** | **SE** | **T score** | **P** |
| --- | --- | --- | --- | --- | --- |
| Felt hated by family member as a child | The frequency of alcohol drinking | 0.08(0.05~0.11) | 0.02 | 4.66 | 3.10e-06 |
| Felt hated by family member as a child | The frequency of cigarette smoking | 0.42(0.40~0.45) | 0.02 | 26.20 | <1e-9 |
| Felt loved as a child | The frequency of alcohol drinking | -0.06(-0.08~-0.04) | 0.01 | -5.11 | 3.15e-07 |
| Felt loved as a child | The frequency of cigarette smoking | -0.31(-0.33~-0.29) | 0.01 | -27.52 | <1e-9 |
| Sexually molested as a child | The frequency of alcohol drinking | 0.06(0.00~0.11) | 0.03 | 1.81 | 0.07 |
| Sexually molested as a child | The frequency of cigarette smoking | 0.46(0.41~0.50) | 0.03 | 16.30 | <1e-9 |
