# Supplementary material for: Traumatic events during childhood and its risks to substance use in adulthood: an observational and genome-wide by environment interaction study in UK Biobank
Source: Transl Psychiatry. 2021 Aug 20;11:431. doi: 10.1038/s41398-021-01557-7 (PMC8379203; doi:10.1038/s41398-021-01557-7)
Supplement: Supplementary file 2 — Interactions between individual SNPs and felt hated by family member as a child in the frequency of cigarette smoking with P <5×10^–8^. [file 41398_2021_1557_MOESM2_ESM.docx]

**Table S2. Interactions between individual SNPs and felt hated by family member as a child in the frequency of cigarette smoking with P <5×10^–8^.**

| **Chromosome** | **Position** | **SNP** | **Beta** | **SE** | **P** |
| --- | --- | --- | --- | --- | --- |
| 9 | 136385554 | rs72779234 | 1.0220 | 0.1763 | 6.81E-09 |
| 6 | 139827777 | rs633417 | 0.6549 | 0.1134 | 7.82E-09 |
| 4 | 65515037 | rs1425386 | 0.5431 | 0.0965 | 1.86E-08 |
| 4 | 65513724 | rs1425389 | -0.5427 | 0.0967 | 2.01E-08 |
| 4 | 65512037 | rs6847676 | 0.5424 | 0.0967 | 2.06E-08 |
| 4 | 65512637 | rs10006240 | 0.5423 | 0.0967 | 2.06E-08 |
| 4 | 65517439 | rs10028130 | 0.5417 | 0.0967 | 2.11E-08 |
| 4 | 65518385 | rs1991220 | 0.5385 | 0.0965 | 2.42E-08 |
| 4 | 65516841 | rs1364863 | 0.5385 | 0.0966 | 2.52E-08 |
| 4 | 65516465 | rs60908926 | 0.5385 | 0.0966 | 2.53E-08 |
| 4 | 65515160 | rs1425385 | 0.5384 | 0.0967 | 2.56E-08 |
| 4 | 65561196 | rs6551865 | 0.5382 | 0.0968 | 2.71E-08 |
| 4 | 65560911 | rs6825923 | 0.5381 | 0.0968 | 2.72E-08 |
| 4 | 65561413 | rs7670098 | 0.5385 | 0.0969 | 2.73E-08 |
| 4 | 65561387 | rs6551867 | 0.5384 | 0.0969 | 2.74E-08 |
| 4 | 65559285 | rs62313332 | 0.5376 | 0.0968 | 2.79E-08 |
| 4 | 65559194 | rs62313331 | 0.5374 | 0.0968 | 2.83E-08 |
| 4 | 65559670 | rs6844247 | 0.5374 | 0.0968 | 2.86E-08 |
| 4 | 65561957 | rs7674465 | 0.5388 | 0.0973 | 3.06E-08 |
| 4 | 65562649 | rs4073463 | 0.5418 | 0.0980 | 3.28E-08 |
| 4 | 65513565 | rs28704970 | 0.5339 | 0.0966 | 3.29E-08 |
| 4 | 65559409 | rs532055983 | -0.5470 | 0.0995 | 3.92E-08 |
| 10 | 67788369 | rs189142060 | 0.5637 | 0.1028 | 4.23E-08 |
| 10 | 67788370 | rs79801998 | 0.5637 | 0.1028 | 4.23E-08 |
| 10 | 67788364 | rs77054425 | 0.5637 | 0.1028 | 4.23E-08 |
| 10 | 67788384 | rs67925426 | 0.5637 | 0.1028 | 4.24E-08 |
| 4 | 65562987 | rs4308391 | 0.5399 | 0.0985 | 4.26E-08 |
