# Supplementary material for: Traumatic events during childhood and its risks to substance use in adulthood: an observational and genome-wide by environment interaction study in UK Biobank
Source: Transl Psychiatry. 2021 Aug 20;11:431. doi: 10.1038/s41398-021-01557-7 (PMC8379203; doi:10.1038/s41398-021-01557-7)
Supplement: Supplementary file 3 — Interactions between individual SNPs and felt hated by family member as a child in the frequency of alcohol drinking with P <5×10^–8^. [file 41398_2021_1557_MOESM3_ESM.docx]

**Table S3.** **Interactions between individual SNPs and felt hated by family member as a child in the frequency of alcohol drinking with P <5×10^–8^.**

| **Chromosome** | **Position** | **SNP** | **Beta** | **SE** | **P** |
| --- | --- | --- | --- | --- | --- |
| 15 | 95399032 | rs184779992 | 2.9310 | 0.4555 | 1.25E-10 |
| 17 | 63737758 | rs186204465 | 3.3107 | 0.5153 | 1.33E-10 |
| 17 | 63788288 | rs112914905 | 3.1650 | 0.5068 | 4.27E-10 |
| 19 | 28129089 | rs575005923 | 2.2146 | 0.3550 | 4.45E-10 |
| 5 | 140888653 | rs147084289 | 2.8942 | 0.4775 | 1.36E-09 |
| 11 | 121987093 | rs17246395 | 1.4772 | 0.2447 | 1.59E-09 |
| 8 | 1617627 | rs138412709 | 2.9509 | 0.4893 | 1.64E-09 |
| 10 | 7320946 | rs145009935 | 3.2660 | 0.5438 | 1.91E-09 |
| 6 | 33856788 | rs143738752 | 3.4558 | 0.5852 | 3.53E-09 |
| 4 | 71886937 | rs542449847 | 3.0218 | 0.5150 | 4.46E-09 |
| 2 | 160877169 | rs116708930 | 3.1571 | 0.5388 | 4.66E-09 |
| 12 | 118207579 | rs61937726 | 2.0721 | 0.3581 | 7.24E-09 |
| 13 | 28236975 | rs492553 | 0.7834 | 0.1355 | 7.50E-09 |
| 12 | 56669799 | rs61738833 | 2.0522 | 0.3553 | 7.66E-09 |
| 4 | 128361185 | rs34481291 | 0.7610 | 0.1320 | 8.13E-09 |
| 17 | 61438429 | rs117358906 | 2.0003 | 0.3488 | 9.77E-09 |
| 11 | 122042785 | rs55783227 | 1.4294 | 0.2494 | 9.94E-09 |
| 10 | 74081232 | rs145028791 | 3.0897 | 0.5391 | 1.00E-08 |
| 16 | 18579524 | rs540601893 | 3.2093 | 0.5625 | 1.17E-08 |
| 4 | 89518459 | rs28549240 | 1.7951 | 0.3161 | 1.36E-08 |
| 4 | 127577100 | rs542533021 | 2.2841 | 0.4025 | 1.39E-08 |
| 8 | 69467874 | rs118114209 | 2.2456 | 0.3971 | 1.57E-08 |
| 11 | 121999548 | rs17337565 | 1.3474 | 0.2391 | 1.76E-08 |
| 2 | 160579578 | rs115125730 | 2.9686 | 0.5271 | 1.79E-08 |
| 11 | 40983189 | rs183497996 | 2.6890 | 0.4778 | 1.84E-08 |
| 11 | 3134196 | rs72844075 | 1.8395 | 0.3284 | 2.14E-08 |
| 8 | 74230168 | rs534525861 | 2.5651 | 0.4582 | 2.17E-08 |
| 11 | 80326287 | rs117763281 | 1.7865 | 0.3197 | 2.30E-08 |
| 5 | 101915290 | rs148104955 | 2.6306 | 0.4710 | 2.35E-08 |
| 4 | 127650160 | rs150252798 | 2.0301 | 0.3638 | 2.42E-08 |
| 15 | 27231451 | rs117020886 | 2.0669 | 0.3720 | 2.77E-08 |
| 2 | 173026855 | rs115126995 | 2.0207 | 0.3639 | 2.83E-08 |
| 17 | 5596725 | rs150899893 | 2.1372 | 0.3855 | 2.98E-08 |
| 9 | 101324654 | rs75210337 | 1.7424 | 0.3144 | 3.01E-08 |
| 4 | 189319695 | rs146081732 | 2.1079 | 0.3804 | 3.01E-08 |
| 4 | 189319054 | rs140112948 | 2.1034 | 0.3800 | 3.12E-08 |
| 9 | 8363231 | rs77261378 | 2.3067 | 0.4180 | 3.44E-08 |
| 7 | 76574906 | rs113905759 | 2.0509 | 0.3731 | 3.87E-08 |
| 17 | 13890109 | rs76609685 | 2.4127 | 0.4401 | 4.22E-08 |
| 19 | 28108958 | rs118051372 | 1.9716 | 0.3599 | 4.31E-08 |
| 17 | 12626614 | rs72811298 | 2.5983 | 0.4745 | 4.38E-08 |
| 21 | 38030577 | rs192955819 | 4.2186 | 0.7724 | 4.74E-08 |
