# Supplementary material for: Traumatic events during childhood and its risks to substance use in adulthood: an observational and genome-wide by environment interaction study in UK Biobank
Source: Transl Psychiatry. 2021 Aug 20;11:431. doi: 10.1038/s41398-021-01557-7 (PMC8379203; doi:10.1038/s41398-021-01557-7)
Supplement: Supplementary file 4 — Interactions between individual SNPs and felt loved as a child in the frequency of cigarette smoking with P <5×10^–8^. [file 41398_2021_1557_MOESM4_ESM.docx]

**Table S4. Interactions between individual SNPs and** **felt loved as a child in the frequency of cigarette smoking with P <5×10^–8^.**

| **Chromosome** | **Position** | **SNP** | **Beta** | **SE** | **P** |
| --- | --- | --- | --- | --- | --- |
| 4 | 138877722 | rs75275910 | -1.0821 | 0.1832 | 3.48E-09 |
| 11 | 1897836 | rs80005225 | -1.9789 | 0.3370 | 4.31E-09 |
| 14 | 37989884 | rs143458035 | -2.2825 | 0.3960 | 8.28E-09 |
| 4 | 138862790 | rs138150520 | -0.9575 | 0.1687 | 1.40E-08 |
| 4 | 138850686 | rs112974254 | -0.9572 | 0.1688 | 1.42E-08 |
| 4 | 138862726 | rs114641798 | -0.9569 | 0.1687 | 1.43E-08 |
| 4 | 138850930 | rs74433654 | -0.9566 | 0.1688 | 1.45E-08 |
| 4 | 138849199 | rs78082518 | -0.9563 | 0.1688 | 1.46E-08 |
| 6 | 146583120 | rs73009056 | -0.9806 | 0.1733 | 1.53E-08 |
| 4 | 138890614 | rs75477682 | -0.9511 | 0.1684 | 1.64E-08 |
| 4 | 138864999 | rs79165613 | -0.9559 | 0.1694 | 1.69E-08 |
| 4 | 138864374 | rs144365439 | -0.9520 | 0.1688 | 1.70E-08 |
| 4 | 138888813 | rs529096243 | -0.9502 | 0.1688 | 1.82E-08 |
| 4 | 138877722 | rs115156713 | -0.9422 | 0.1691 | 2.52E-08 |
| 4 | 138860199 | rs35746154 | -0.9460 | 0.1700 | 2.65E-08 |
| 4 | 138867152 | rs116549693 | -0.9418 | 0.1696 | 2.79E-08 |
| 4 | 138887240 | rs114560683 | -0.9352 | 0.1684 | 2.79E-08 |
| 4 | 138842236 | rs77600838 | -0.9416 | 0.1696 | 2.83E-08 |
| 4 | 138882901 | rs115324076 | -0.9414 | 0.1696 | 2.85E-08 |
| 4 | 138884672 | rs74879070 | -0.9383 | 0.1690 | 2.86E-08 |
| 4 | 138844944 | rs75349989 | -0.9420 | 0.1697 | 2.87E-08 |
| 4 | 138867950 | rs113147440 | -0.9336 | 0.1684 | 2.98E-08 |
| 4 | 138833878 | rs77973786 | -0.9408 | 0.1697 | 2.98E-08 |
| 4 | 138878187 | rs138993402 | -0.9329 | 0.1683 | 3.00E-08 |
| 4 | 138836311 | rs116636119 | -0.9329 | 0.1683 | 3.00E-08 |
| 4 | 138836792 | rs113544708 | -0.9324 | 0.1683 | 3.05E-08 |
| 4 | 138833880 | rs114508782 | -0.9400 | 0.1697 | 3.06E-08 |
| 4 | 138874101 | rs78815960 | -0.9396 | 0.1697 | 3.10E-08 |
| 4 | 138872394 | rs77773972 | -0.9316 | 0.1683 | 3.13E-08 |
| 4 | 138832512 | rs76534207 | -0.9392 | 0.1697 | 3.15E-08 |
| 1 | 138875179 | rs116127176 | -2.0299 | 0.3702 | 4.19E-08 |
