# Supplementary material for: Traumatic events during childhood and its risks to substance use in adulthood: an observational and genome-wide by environment interaction study in UK Biobank
Source: Transl Psychiatry. 2021 Aug 20;11:431. doi: 10.1038/s41398-021-01557-7 (PMC8379203; doi:10.1038/s41398-021-01557-7)
Supplement: Supplementary file 5 — Interactions between individual SNPs and felt loved as a child in the frequency of alcohol drinking with P <5×10^–8^. [file 41398_2021_1557_MOESM5_ESM.docx]

**Table S5. Interactions between individual SNPs and felt loved as a child in the frequency of alcohol drinking with P <5×10^–8^.**

| **Chromosome** | **Position** | **SNP** | **Beta** | **SE** | **P** |
| --- | --- | --- | --- | --- | --- |
| 1 | 188642561 | rs76704560 | -2.7687 | 0.3937 | 2.04E-12 |
| 13 | 109922854 | rs188250047 | 0.6294 | 0.0979 | 1.29E-10 |
| 11 | 37536125 | rs138680314 | -2.6344 | 0.4161 | 2.46E-10 |
| 6 | 56342136 | rs34892827 | -1.6300 | 0.2625 | 5.34E-10 |
| 16 | 56861543 | rs79382425 | -1.2116 | 0.1999 | 1.36E-09 |
| 16 | 56803025 | rs78087962 | -1.1865 | 0.1971 | 1.74E-09 |
| 14 | 58295587 | rs147222280 | -2.1167 | 0.3533 | 2.09E-09 |
| 11 | 102500833 | rs2846344 | -0.5446 | 0.0915 | 2.67E-09 |
| 8 | 69064260 | rs62522696 | -1.6538 | 0.2784 | 2.87E-09 |
| 1 | 216393301 | rs142253038 | -2.1776 | 0.3671 | 3.02E-09 |
| 17 | 8952612 | rs79795728 | -2.1540 | 0.3634 | 3.08E-09 |
| 14 | 59082662 | rs74326053 | -1.8907 | 0.3192 | 3.16E-09 |
| 14 | 59041283 | rs115882441 | -1.7505 | 0.2960 | 3.38E-09 |
| 11 | 102500807 | rs2846345 | -0.5389 | 0.0915 | 3.90E-09 |
| 14 | 59037440 | rs76924950 | -1.7400 | 0.2956 | 3.95E-09 |
| 11 | 102501507 | rs2846358 | -0.5375 | 0.0913 | 3.98E-09 |
| 11 | 102500813 | rs2701975 | -0.5378 | 0.0915 | 4.19E-09 |
| 10 | 123685956 | rs4752622 | -1.2061 | 0.2054 | 4.31E-09 |
| 10 | 123696172 | rs11200272 | -1.1960 | 0.2045 | 4.97E-09 |
| 19 | 17706896 | rs11878930 | -1.0890 | 0.1865 | 5.24E-09 |
| 1 | 30066051 | rs7544811 | -0.5000 | 0.0857 | 5.34E-09 |
| 2 | 108827987 | rs76304485 | -1.5736 | 0.2697 | 5.43E-09 |
| 11 | 102499007 | rs1320659 | -0.5256 | 0.0910 | 7.66E-09 |
| 2 | 242488954 | rs116596424 | -1.8097 | 0.3136 | 7.92E-09 |
| 11 | 102493335 | rs1784404 | -0.5239 | 0.0908 | 8.03E-09 |
| 11 | 102500093 | rs2701971 | -0.5265 | 0.0913 | 8.04E-09 |
| 14 | 58937452 | rs78511804 | -1.7996 | 0.3123 | 8.36E-09 |
| 11 | 102500577 | rs2701974 | -0.5261 | 0.0913 | 8.46E-09 |
| 11 | 43607183 | rs145993662 | -2.1317 | 0.3702 | 8.51E-09 |
| 11 | 102499440 | rs2701969 | -0.5235 | 0.0910 | 8.93E-09 |
| 7 | 149881668 | rs10251233 | -1.2196 | 0.2121 | 8.94E-09 |
| 11 | 102496462 | rs2245897 | -0.5232 | 0.0910 | 9.00E-09 |
| 4 | 134883971 | rs4398581 | -1.5315 | 0.2664 | 9.03E-09 |
| 11 | 102496800 | rs2459474 | -0.5231 | 0.0910 | 9.10E-09 |
| 11 | 102497070 | rs2464356 | -0.5230 | 0.0910 | 9.13E-09 |
| 11 | 102497310 | rs1940050 | -0.5230 | 0.0910 | 9.16E-09 |
| 11 | 102497164 | rs2464357 | -0.5229 | 0.0910 | 9.18E-09 |
| 11 | 102497237 | rs2464358 | -0.5229 | 0.0910 | 9.21E-09 |
| 11 | 102494160 | rs2464352 | -0.5214 | 0.0908 | 9.22E-09 |
| 11 | 102497598 | rs1940049 | -0.5228 | 0.0910 | 9.26E-09 |
| 11 | 102497891 | rs2701965 | -0.5228 | 0.0910 | 9.27E-09 |
| 11 | 102497703 | rs1940047 | -0.5228 | 0.0910 | 9.28E-09 |
| 11 | 102497782 | rs1940046 | -0.5228 | 0.0910 | 9.29E-09 |
| 11 | 102498211 | rs2009489 | -0.5227 | 0.0910 | 9.33E-09 |
| 11 | 102498276 | rs2009487 | -0.5227 | 0.0910 | 9.33E-09 |
| 11 | 102498563 | rs2701966 | -0.5226 | 0.0910 | 9.37E-09 |
| 11 | 102498449 | rs2464359 | -0.5226 | 0.0910 | 9.38E-09 |
| 11 | 102498722 | rs2846347 | -0.5225 | 0.0910 | 9.42E-09 |
| 11 | 102500040 | rs2846346 | -0.5239 | 0.0913 | 9.46E-09 |
| 11 | 102498908 | rs2464360 | -0.5224 | 0.0910 | 9.47E-09 |
| 11 | 102499087 | rs2509024 | -0.5224 | 0.0910 | 9.51E-09 |
| 11 | 102494289 | rs2464354 | -0.5208 | 0.0907 | 9.54E-09 |
| 11 | 102498113 | rs2009659 | -0.5221 | 0.0910 | 9.68E-09 |
| 11 | 102499340 | rs2701967 | -0.5220 | 0.0910 | 9.80E-09 |
| 11 | 102499359 | rs2701968 | -0.5219 | 0.0910 | 9.85E-09 |
| 14 | 59007601 | rs74414016 | -1.7718 | 0.3090 | 9.89E-09 |
| 1 | 216455033 | rs147347821 | -2.1071 | 0.3676 | 1.00E-08 |
| 8 | 12601034 | rs6530964 | 0.4761 | 0.0831 | 1.02E-08 |
| 11 | 102494744 | rs2245697 | -0.5187 | 0.0907 | 1.07E-08 |
| 4 | 168547414 | rs72705325 | -1.3698 | 0.2396 | 1.09E-08 |
| 6 | 147590019 | rs78477794 | -1.7206 | 0.3010 | 1.10E-08 |
| 11 | 102495035 | rs2464355 | -0.5173 | 0.0906 | 1.15E-08 |
| 11 | 102495718 | rs2459476 | -0.5170 | 0.0906 | 1.16E-08 |
| 11 | 102495712 | rs2509022 | -0.5170 | 0.0906 | 1.17E-08 |
| 11 | 102495745 | rs2509023 | -0.5170 | 0.0906 | 1.17E-08 |
| 11 | 102494161 | rs2464353 | -0.5181 | 0.0909 | 1.20E-08 |
| 7 | 155607225 | rs138964452 | -2.3923 | 0.4201 | 1.24E-08 |
| 11 | 102500352 | rs2701973 | -0.5189 | 0.0913 | 1.33E-08 |
| 11 | 102495320 | rs7116246 | -0.5151 | 0.0907 | 1.36E-08 |
| 4 | 75255735 | rs112444088 | -1.6460 | 0.2900 | 1.39E-08 |
| 14 | 59034137 | rs148067634 | -1.6911 | 0.2981 | 1.41E-08 |
| 19 | 45518988 | rs10412986 | -0.5302 | 0.0935 | 1.42E-08 |
| 14 | 88341659 | rs377453624 | -2.1305 | 0.3763 | 1.50E-08 |
| 4 | 118230910 | rs4432822 | -1.7363 | 0.3070 | 1.56E-08 |
| 5 | 79174830 | rs77190514 | -1.0304 | 0.1825 | 1.64E-08 |
| 8 | 59924790 | rs16924419 | -0.7490 | 0.1327 | 1.67E-08 |
| 14 | 59111736 | rs181625218 | -1.7689 | 0.3134 | 1.67E-08 |
| 17 | 37372773 | rs79873275 | -1.0686 | 0.1900 | 1.87E-08 |
| 1 | 240809551 | rs186110361 | -2.1337 | 0.3798 | 1.95E-08 |
| 14 | 32902252 | rs79187523 | -1.6041 | 0.2858 | 2.01E-08 |
| 3 | 28681920 | rs150200887 | -2.2039 | 0.3930 | 2.06E-08 |
| 4 | 156566035 | rs145936829 | -1.7923 | 0.3196 | 2.07E-08 |
| 8 | 59923874 | rs77206554 | -0.7433 | 0.1326 | 2.08E-08 |
| 8 | 142991042 | rs191150200 | -2.1591 | 0.3855 | 2.15E-08 |
| 11 | 102491248 | rs11383572 | -0.5093 | 0.0910 | 2.19E-08 |
| 11 | 102500098 | rs2701972 | -0.5090 | 0.0910 | 2.24E-08 |
| 8 | 59922797 | rs181229803 | -0.7489 | 0.1341 | 2.34E-08 |
| 13 | 39806969 | rs75774241 | -1.5536 | 0.2782 | 2.36E-08 |
| 8 | 59922796 | rs189574629 | -0.7483 | 0.1340 | 2.38E-08 |
| 1 | 30064009 | rs6603927 | -0.4456 | 0.0799 | 2.42E-08 |
| 14 | 58326245 | rs140104819 | -1.8625 | 0.3341 | 2.48E-08 |
| 4 | 122583712 | rs541590009 | -1.5647 | 0.2810 | 2.59E-08 |
| 4 | 122583713 | rs561509989 | -1.5636 | 0.2811 | 2.66E-08 |
| 11 | 102492505 | rs1711405 | -0.5049 | 0.0909 | 2.77E-08 |
| 11 | 102492692 | rs1630876 | -0.5049 | 0.0909 | 2.77E-08 |
| 5 | 161952417 | rs114969140 | -2.1184 | 0.3817 | 2.86E-08 |
| 1 | 216534675 | rs148183513 | -2.1094 | 0.3801 | 2.87E-08 |
| 17 | 37711360 | rs111458598 | -1.0880 | 0.1961 | 2.91E-08 |
| 5 | 117244014 | rs6897293 | -1.7970 | 0.3242 | 2.98E-08 |
| 8 | 59926656 | rs16924429 | -0.7369 | 0.1330 | 3.06E-08 |
| 10 | 13099762 | rs61853201 | -0.5380 | 0.0972 | 3.11E-08 |
| 1 | 30068857 | rs9660251 | -0.4437 | 0.0802 | 3.16E-08 |
| 1 | 55903579 | rs12724160 | -0.5290 | 0.0957 | 3.23E-08 |
| 4 | 156594909 | rs150929669 | -1.7603 | 0.3185 | 3.27E-08 |
| 11 | 102490410 | rs2459477 | -0.5014 | 0.0909 | 3.46E-08 |
| 10 | 13096841 | rs61262702 | -0.5390 | 0.0977 | 3.46E-08 |
| 11 | 102489189 | rs1784414 | -0.5013 | 0.0909 | 3.49E-08 |
| 11 | 102491550 | rs1711406 | -0.5011 | 0.0909 | 3.51E-08 |
| 11 | 102491444 | rs1711407 | -0.5011 | 0.0909 | 3.51E-08 |
| 11 | 102491990 | rs1624909 | -0.5011 | 0.0909 | 3.51E-08 |
| 1 | 39276265 | rs75028545 | -1.4973 | 0.2715 | 3.52E-08 |
| 11 | 102491515 | rs1784402 | -0.5011 | 0.0909 | 3.52E-08 |
| 11 | 102490672 | rs1784401 | -0.5009 | 0.0909 | 3.56E-08 |
| 10 | 51038547 | rs17010387 | -1.4545 | 0.2642 | 3.71E-08 |
| 10 | 13094866 | rs11592430 | -0.5383 | 0.0978 | 3.73E-08 |
| 4 | 134759852 | rs146636827 | -1.6265 | 0.2956 | 3.77E-08 |
| 9 | 78138859 | rs111568486 | -1.8265 | 0.3322 | 3.84E-08 |
| 8 | 59906910 | rs78300448 | -0.7318 | 0.1335 | 4.19E-08 |
| 8 | 59909111 | rs61514480 | -0.7314 | 0.1335 | 4.28E-08 |
| 10 | 51783715 | rs367757185 | -0.5914 | 0.1081 | 4.55E-08 |
| 3 | 131322682 | rs114438632 | -1.3971 | 0.2555 | 4.57E-08 |
| 4 | 122587875 | rs115600322 | -1.5253 | 0.2790 | 4.57E-08 |
| 4 | 122590444 | rs116573968 | -1.5249 | 0.2790 | 4.61E-08 |
| 8 | 59900124 | rs10504272 | -0.7303 | 0.1336 | 4.62E-08 |
| 3 | 167873353 | rs146233927 | -1.5979 | 0.2923 | 4.62E-08 |
| 4 | 122592200 | rs78995989 | -1.5241 | 0.2789 | 4.68E-08 |
| 4 | 122594301 | rs145434780 | -1.5237 | 0.2789 | 4.71E-08 |
| 4 | 122595100 | rs28557003 | -1.5241 | 0.2790 | 4.72E-08 |
| 8 | 59897223 | rs111937104 | -0.7293 | 0.1336 | 4.82E-08 |
| 10 | 13101450 | rs34472855 | -0.5311 | 0.0974 | 4.89E-08 |
| 4 | 122602284 | rs11726034 | -1.5215 | 0.2789 | 4.89E-08 |
| 10 | 51783304 | rs4532987 | -0.5853 | 0.1073 | 4.94E-08 |
| 10 | 13100269 | rs9787488 | -0.5305 | 0.0973 | 4.95E-08 |
