# Supplementary material for: Traumatic events during childhood and its risks to substance use in adulthood: an observational and genome-wide by environment interaction study in UK Biobank
Source: Transl Psychiatry. 2021 Aug 20;11:431. doi: 10.1038/s41398-021-01557-7 (PMC8379203; doi:10.1038/s41398-021-01557-7)
Supplement: Supplementary file 6 — Interactions between individual SNPs and sexually molested as a child in the frequency of cigarette smoking with P <5×10^–8^. [file 41398_2021_1557_MOESM6_ESM.docx]

**Table S6. Interactions between individual SNPs and sexually molested as a child in the frequency of cigarette smoking with P <5×10^–8^.**

| **Chromosome** | **Position** | **SNP** | **Beta** | **SE** | **P** |
| --- | --- | --- | --- | --- | --- |
| 18 | 20131590 | rs113124813 | 1.3799 | 0.2233 | 6.46E-10 |
| 18 | 20119264 | rs111885617 | 1.3899 | 0.2269 | 9.06E-10 |
| 10 | 30102709 | rs141708820 | 1.3747 | 0.2252 | 1.04E-09 |
| 9 | 76733681 | rs10119625 | 0.8523 | 0.1428 | 2.44E-09 |
| 9 | 76724988 | rs4436184 | 0.8631 | 0.1455 | 2.98E-09 |
| 9 | 76719502 | rs4263837 | 0.8752 | 0.1478 | 3.19E-09 |
| 21 | 46924322 | rs116618591 | 1.9578 | 0.3307 | 3.23E-09 |
| 9 | 76734711 | rs7024222 | 0.8557 | 0.1450 | 3.66E-09 |
| 18 | 20072399 | rs111805396 | 1.4280 | 0.2421 | 3.67E-09 |
| 9 | 105081995 | rs550801461 | 1.7006 | 0.2908 | 5.00E-09 |
| 4 | 91906746 | rs200062414 | 1.3670 | 0.2342 | 5.35E-09 |
| 9 | 76717403 | rs4520234 | 0.8728 | 0.1500 | 5.95E-09 |
| 2 | 57646507 | rs11676537 | 2.5464 | 0.4396 | 6.95E-09 |
| 11 | 19276917 | rs78596622 | 1.5519 | 0.2702 | 9.29E-09 |
| 2 | 57707193 | rs75086379 | 2.9496 | 0.5139 | 9.54E-09 |
| 4 | 55277918 | rs112497592 | 1.1471 | 0.2006 | 1.08E-08 |
| 14 | 79663898 | rs564134655 | 0.4820 | 0.0848 | 1.34E-08 |
| 20 | 1228183 | rs62186523 | 2.0057 | 0.3549 | 1.60E-08 |
| 3 | 172534977 | rs75052594 | 2.3871 | 0.4226 | 1.62E-08 |
| 4 | 186532425 | rs115349299 | 1.3142 | 0.2328 | 1.65E-08 |
| 1 | 57650410 | rs58359668 | 0.8037 | 0.1425 | 1.69E-08 |
| 1 | 57648856 | rs17115257 | 0.8029 | 0.1424 | 1.72E-08 |
| 14 | 94965837 | rs17825787 | 1.3464 | 0.2392 | 1.83E-08 |
| 9 | 5707663 | rs552868666 | 0.9579 | 0.1708 | 2.04E-08 |
| 8 | 104659643 | rs75600780 | 2.0832 | 0.3736 | 2.48E-08 |
| 6 | 6796474 | rs144852671 | 1.5539 | 0.2788 | 2.51E-08 |
| 8 | 104658315 | rs147591966 | 2.0814 | 0.3737 | 2.55E-08 |
| 8 | 104839791 | rs149853430 | 2.0777 | 0.3737 | 2.70E-08 |
| 8 | 104826892 | rs149185881 | 2.0776 | 0.3736 | 2.70E-08 |
| 2 | 44284924 | rs10202948 | -0.4527 | 0.0815 | 2.84E-08 |
| 21 | 46927999 | rs374847238 | 1.8304 | 0.3301 | 2.94E-08 |
| 8 | 54045826 | rs2376427 | -0.3886 | 0.0701 | 2.99E-08 |
| 8 | 104557045 | rs151099034 | 2.0700 | 0.3741 | 3.15E-08 |
| 8 | 54097832 | rs2553907 | -0.3885 | 0.0702 | 3.16E-08 |
| 9 | 5835362 | rs13300893 | 0.9404 | 0.1700 | 3.18E-08 |
| 8 | 54095563 | rs2717650 | -0.3884 | 0.0702 | 3.19E-08 |
| 8 | 54096391 | rs71252982 | -0.3879 | 0.0703 | 3.38E-08 |
| 14 | 79448958 | rs183288119 | 0.9029 | 0.1637 | 3.47E-08 |
| 8 | 104619998 | rs539524469 | 2.2903 | 0.4157 | 3.62E-08 |
| 1 | 176789615 | rs76808343 | 1.0085 | 0.1832 | 3.72E-08 |
| 3 | 172542427 | rs145181957 | 2.2632 | 0.4120 | 3.98E-08 |
| 8 | 54045562 | rs2553852 | -0.3839 | 0.0700 | 4.09E-08 |
| 8 | 54046946 | rs2588075 | -0.3838 | 0.0699 | 4.09E-08 |
| 8 | 54048390 | rs2717618 | -0.3837 | 0.0699 | 4.10E-08 |
| 8 | 54047269 | rs969078 | -0.3838 | 0.0699 | 4.10E-08 |
| 8 | 54047711 | rs1425916 | -0.3837 | 0.0699 | 4.10E-08 |
| 8 | 54047411 | rs969077 | -0.3838 | 0.0699 | 4.11E-08 |
| 8 | 54049277 | rs111386099 | -0.3833 | 0.0699 | 4.14E-08 |
| 8 | 54046044 | rs1895834 | -0.3835 | 0.0699 | 4.19E-08 |
| 8 | 54049026 | rs1834480 | -0.3833 | 0.0699 | 4.21E-08 |
| 8 | 54048643 | rs2588074 | -0.3832 | 0.0699 | 4.27E-08 |
| 15 | 97903174 | rs80121476 | 2.1680 | 0.3965 | 4.59E-08 |
| 3 | 184963221 | rs114730935 | 2.3927 | 0.4385 | 4.87E-08 |
| 8 | 98913844 | rs77983918 | 1.0065 | 0.1846 | 4.95E-08 |
